# Nature of Fast Relaxation Processes and Spectroscopy of a Membrane-Active Peptide Modified with Fluorescent Amino Acid Exhibiting Excited State Intramolecular Proton Transfer and Efficient Stimulated Emission

**DOI:** 10.1021/acsomega.1c00193

**Published:** 2021-04-05

**Authors:** Yevgeniy O. Shaydyuk, Nataliia V. Bashmakova, Andriy M. Dmytruk, Olexiy D. Kachkovsky, Serhii Koniev, Alexander V. Strizhak, Igor V. Komarov, Kevin D. Belfield, Mykhailo V. Bondar, Oleg Babii

**Affiliations:** † Institute of Physics National Academy of Sciences of Ukraine, Prospect Nauki 46, Kyiv 03028, Ukraine; ‡ Taras Shevchenko National University of Kyiv, Volodymyrska Street 60, Kyiv 01601, Ukraine; § V.P. Kukhar Institute of Bioorganic Chemistry and Petrochemistry of the National Academy of Sciences, Murmanskaya Street 1, Kyiv 02660, Ukraine; ∥ Enamine Ltd, Vul. Chervonotkatska 78, Kyiv 02094, Ukraine; ⊥ New Jersey Institute of Technology, College of Science and Liberal Arts, University Heights, Newark, New Jersey 07102, United States; # Institute of Biological Interfaces (IBG-2), Karlsruhe Institute of Technology (KIT), POB3640, Karlsruhe 76021, Germany

## Abstract

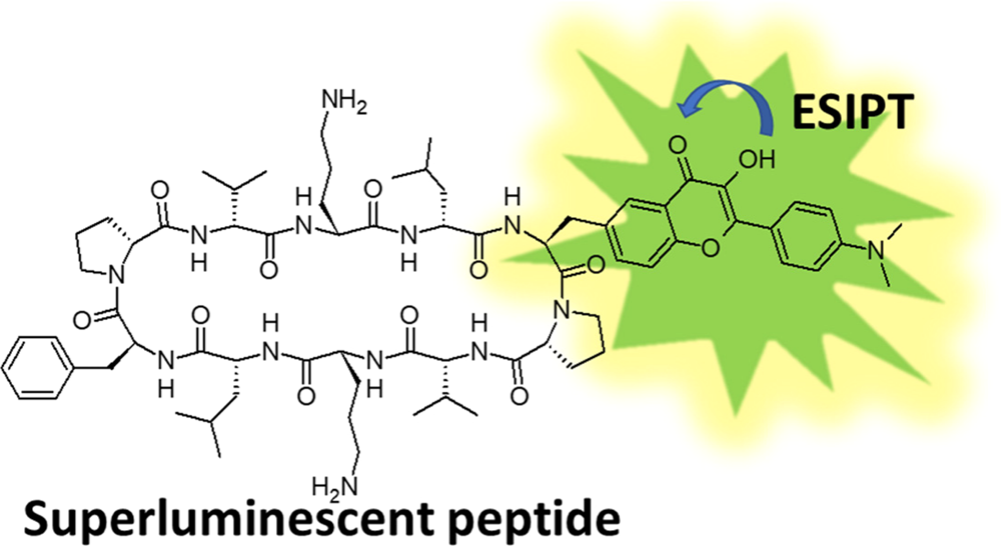

A fluorescently labeled peptide that exhibited fast excited state intramolecular proton transfer (ESIPT) was synthesized, and the nature of its electronic properties was comprehensively investigated, including linear photophysical and photochemical characterization, specific relaxation processes in the excited state, and its stimulated emission ability. The steady-state absorption, fluorescence, and excitation anisotropy spectra, along with fluorescence lifetimes and emission quantum yields, were obtained in liquid media and analyzed based on density functional theory quantum-chemical calculations. The nature of ESIPT processes of the peptide’s chromophore moiety was explored using a femtosecond transient absorption pump-probe technique, revealing relatively fast ESIPT velocity (∼10 ps) in protic MeOH at room temperature. Efficient superluminescence properties of the peptide were realized upon femtosecond excitation in the main long-wavelength absorption band with a corresponding threshold of the pump pulse energy of ∼1.5 μJ. Quantum-chemical analysis of the electronic structure of the peptide was performed using the density functional theory/time-dependent density functional theory level of theory, affording good agreement with experimental data.

## 1 Introduction

The synthesis and characterization of new environmentally sensitive fluorescently labeled peptides are of great interest for a broad range of fundamental and applied research fields, including protein–protein and peptide–oligonucleotide interactions,^1,2^ dynamics of peptide binding,^3,4^ ion sensing,^5,6^ pH monitoring,^7,8^ and fluorescence cellular bioimaging.^9,10^ The fluorescence characteristics of a peptide’s emission are primarily determined by the properties of the chromophore moiety incorporated into the peptide structure^11,12^ and can serve as a starting point in the development of corresponding applications mentioned above. One of the promising classes of chromophore systems that can be used in peptide structural context are those that exhibit excited state intramolecular proton transfer (ESIPT),^13,14^ which essentially extends the application potential of fluorescent peptides and proves to be an efficient probe to study peptides in their natural environment.^15−17^

A broad variety of ESIPT chromophores have been reported for use in the labeling of peptides, including 3-hydroxychromone (3HC) derivatives,^15^ 3-hydroxyflavone fluorophores (3HF),^9,16^ benzothiophene-substituted chromenone (CHBT),^18^ and 2-(5′-chloro-2-hydroxyl-phenyl)-6-chloro-4-(3H)-quinazolinone (CHCQ),^19^ just to mention a few. The specificity of ESIPT processes in chromophore structures was comprehensively described in the scientific literature^20−25^ and can be used as a part of specially designed electronic mechanisms for amplified spontaneous emission,^26^ bulk heterojunction solar cells,^27^ color-specific photoswitching,^28^ light-emitting liquid crystal displays,^29^ thermally activated delayed fluorescence,^30^ and so forth. The dynamics of ESIPT phenomena is also an area of great interest,^23,31,32^ and the nature of ultrafast and relatively long proton transfer processes was comprehensively investigated using transient absorption pump-probe spectroscopy,^33−36^ upconverted and time-resolved fluorescence methods,^37,38^ and femtosecond time-resolved resonance-enhanced multiphoton ionization and ion yield spectroscopy techniques.^39,40^

**Figure 1:**
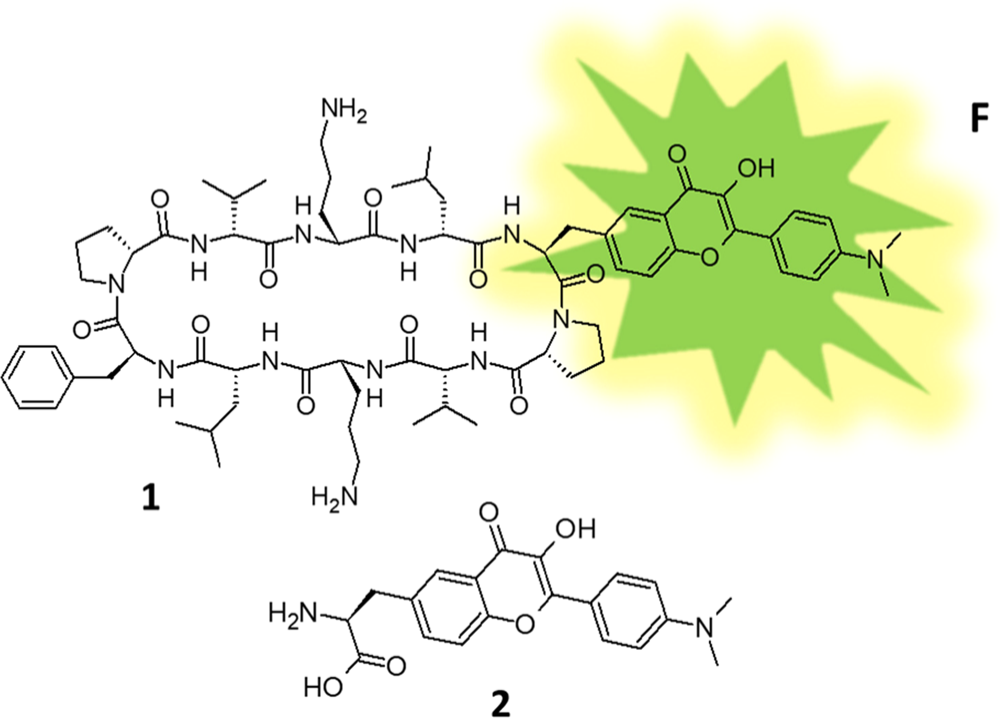
Structural formula of peptide **1** with the fluorophore (**F**) and the noncanonical amino acid **2**, which was used in the synthesis of **1**.

In this work, we present the synthesis and comprehensive investigation of linear steady-state and time-resolved photophysical properties, along with femtosecond transient absorption pump-probe spectroscopy of a new fluorescently labeled peptide **1**, which exhibited the ESIPT phenomenon in liquid media at room temperature and efficient superluminescence under femtosecond pumping into the main absorption band. Peptide **1** (Figure 1) is an analogue of a well-known antimicrobial peptidic antibiotic gramicidin S (*cyclo*[VOLfP]_2_ (O, ornithine; f, *D*-phenylalanine). Although this peptide was discovered more than 75 years ago,^41^ its mechanism of action is still under study and is relevant to its therapeutic applications.^42^ Incorporation of a fluorescent label into the gramicidin S molecule would provide a valuable tool for its study, in particular, in living cells and tissues.

We used the 3HF-derived amino acid **2** for the labeling of gramicidin S (Figure 1), which has already demonstrated its excellent performance in the peptide field.^16^ It is also relevant to note that we used the natural amino acids with an inverted stereo-configuration at the α-carbon atoms (as compared to natural gramicidin S), and the fluorescent label was introduced into the molecule in place of one of the phenylalanine residues. As further studies of this compound can be envisioned to be performed in living organisms, the “inverted” structure may enhance its proteolytic stability.^43^

In this study, we aimed at elucidating further details on the photophysical characteristics of the label to expand the knowledge base and its utility. Specific features in the linear spectral properties of **1** were shown, and the characteristic times of ESIPT processes were determined using a femtosecond pump-probe spectroscopy technique. Density functional theory/time-dependent density functional theory (DFT/TD-DFT) quantum-chemical calculations of the electronic parameters of the normal and tautomeric forms of the chromophore moiety in the peptide were performed, and good agreement with experimental parameters was obtained.

## 2 Experimental Section

### 2.1 Synthesis of the Peptide 1 and Linear Photophysical and Photochemical Characterization

#### 2.1.1 Chemical Synthesis, General

All chemicals and solvents were purchased from Sigma-Aldrich, Iris Biotech, ABCR, Fisher, Carl Roth, and Biosolve. The noncanonical amino acid **2** (3'-(2-[4-(dimethyllamino)phenyl]-3-hydroxy-4-oxo-4H-chromen-6-yl)-*L*-alanine) was synthesized and converted to the N-Fmoc derivative according to the published procedures, using *L*-tyrosine as the starting compound.^16^ Reversed-phase high-performance liquid chromatography (RP-HPLC) analysis for the new compound was performed on a Jasco system equipped with a diode array detector. The following columns and eluting conditions were employed for the peptide: Vydac (218TP) C18 (4.6 mm × 250 mm); column temperature, 40 °C; and flow rate, 1.5 mL/min for analytical high-performance liquid chromatography (HPLC). Vydac (218TP) C18 (22 mm × 250 mm); column temperature, 40 °C; flow rate, 17 mL/min for preparative HPLC. Eluent A: 90% H_2_O, 10% acetonitrile, and 5 mM HCl. Eluent B: 10% H_2_O, 90% acetonitrile, and 5 mM HCl. Gradient slopes of 1 and 4% eluent B/min for analytical and preparative HPLC were used, respectively. According to the HPLC analysis, peptide **1** was ≥95% pure (UV detection, 215 nm). Analytical ^1^H NMR spectra for the N-Fmoc **2** and the intermediates of its synthesis were recorded on a Bruker Avance 400 spectrometer and referenced to tetramethylsilane. The mass spectrum for the peptide identification was recorded on a Bruker Autoflex III instrument, using matrix-assisted laser desorption ionization-time-of-flight (MALDI-TOF) mass spectrometry. Analytical samples were cocrystallized on a Bruker stainless steel target with a matrix of 3,5-dihydroxybenzoic acid or α-cyano-4-hydroxycinnamic acid from acidic water/acetonitrile solutions.

#### 2.1.2 Peptide Synthesis, Purification, and Characterization

Peptide **1** was synthesized by a solid-phase peptide synthesis protocol.^44^ First, the linear sequence was synthesized on a 2-chlorotrityl resin, preloaded with the first amino acid, Fmoc-*D*-leucine. Typical resin load was 0.5–0.8 mmol/g; the reaction scale was 0.2 mmol. The double-coupling protocol (20 min/coupling step) with 4 equiv was set up on an automatic peptide synthesizer Biotage Syro II in the case of N-Fmoc-protected natural amino acids, which were activated in all cases with HBTU and HOBt using DIPEA in DMF. The natural amino acids had a *D*-stereo-configuration at the α-carbon atoms, except the phenylalanine, which had an *L*-configuration. Coupling of the noncanonical amino acid **2** (*L*-configuration, the last in the linear sequence) was performed manually using 1.2 equiv of the Fmoc-protected amino acid, activated with 1.2 equiv of PyAOP (7-azabenzotriazol-1-yloxy)tripyrrolidinophosphonium hexafluorophosphate) and 2.4 equiv of DIPEA in DMF (0.5 mL DMF per 0.1 mmol of the Fmoc-protected **2**).

Fmoc deprotection in all cases was performed with 20% piperidine (20 min in DMF). After completion of the linear sequence, the resin was washed with DCM and dried under vacuum. The linear precursor was cleaved from the resin without side chain deprotection using a mixture of 1,1,1,3,3,3-hexafluoro-2-propanol and DCM (1:3, v/v; 10 mL; and 15 min). The solution was filtered from the resin and dried using a rotary evaporator. The obtained oil was suspended in an acetonitrile/water mixture (1:1, v/v) and lyophilized. The crude linear precursor was used for the cyclization without further purification. The cyclization step was conducted in DCM (0.8 L per 0.2 mmol load) with the activating mixture of PyAOP (2 equiv) predissolved in DMF (2 mL) followed by addition of DIPEA (4 equiv). The reaction mixture was stirred for 18 h. Afterward, the solvent was evaporated on a rotary evaporator, and the residual material was lyophilized. The final deprotection of the cyclized peptide was accomplished with a deprotecting cocktail containing trifluoroacetic acid, triisopropylsilane, and water (92.5:2.5:5, v/v/v and 10 mL) and by incubating for 30 min at room temperature. The volatile products were removed on a rotary evaporator, and the residual oil was lyophilized. The crude peptide was dissolved in 10 mL of water/acetonitrile mixture (2:1, v/v) and analyzed on analytical RP-HPLC. Individual peak fractions from analytical RP-HPLC were collected and analyzed by MALDI-TOF mass spectrometry. The major component in the crude material was confirmed to be the target product. The peptide was purified on a preparative RP-HPLC with a method exploiting a gradient of 30–50% eluent B. The final yield of peptide **1** was 45 mg with purity ≥95%, confirmed by analytical RP-HPLC and MALDI-TOF mass spectrometry. The structural formula of **1** is shown in Figure 1.

#### 2.1.3 Linear Photophysical and Photochemical Characterization

The investigation of **1** was performed in air-saturated acetonitrile (ACN) and methanol (MeOH) at room temperature. All solvents were of spectroscopic-grade, purchased from commercial sources, and used without further purification. The steady-state linear absorption spectra were obtained with a UV–visible spectrophotometer (Shimadzu 2450) using 1 cm path length spectrophotometric quartz cuvettes with compound concentrations, C ∼ (5–7)·10^–5^ M. The steady-state fluorescence, excitation, and excitation anisotropy spectra were measured in standard 1 cm path length spectrofluorometric quartz cuvettes using spectrofluorimeter CM 2203 (Solar, Belarus) and low concentrated solutions (C ∼10^–6^ M) to avoid reabsorption effects.^45^ All emission spectra were corrected for the spectral responsivity of the spectrofluorimeter’s detection system. Fluorescence quantum yields, Φ_fl_, were measured in dilute solution using a standard relative method with 9,10-diphenylanthracene in cyclohexane as a reference.^45^ The steady-state excitation anisotropy spectrum was determined using an “L-format” configuration geometry in viscous medium (glycerol at room temperature), where the molecular rotational correlation time, θ, dramatically exceeds its fluorescence lifetime, τ_fl_, and excitation anisotropy, *r*(λ) = *r*_0_(λ)/(1 + τ_fl_/θ), is nearly equal to its fundamental value, *r*_0_(λ).^45^ The values of fluorescence lifetimes, τ_fl_, were measured with a Life Spec-II spectrometer (Edinburgh Instruments Ltd) in 1 cm path length standard spectrofluorimetric quartz cuvettes and dilute solution.

The investigation of the photochemical stability of **1** was based on the quantitative determination of its photodecomposition quantum yields, Φ_ph_, in different media using an absorption method previously described in detail.^46^ The value of Φ_ph_ is defined as *N*_m_/*N*_hv_ (*N*_m_ and *N*_hv_ are the number of photobleached molecules and absorbed photons, correspondingly) and was determined with the use of the equation:^46^
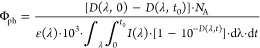
1where *D*(λ,0), *D*(λ, *t*_0_), λ, *N*_A_, ε(λ), and *t*_0_ are the initial and final absorbance of the sample solution, excitation wavelength (cm), Avogadro’s number, extinction coefficient (M^–1^·cm^–1^), and irradiation time (s), respectively; *I*(λ) is the excitation irradiance per unit wavelength (photon·sm^–3^·s^–1^). A light-emitting diode with λ ≈ 405 nm and average beam irradiance ≈ 40 mW/cm^2^ was used as a radiation source.

### 2.2 Transient Absorption Femtosecond Pump-Probe and Superluminescence Measurements

Ultrafast relaxation processes and time-resolved excited state absorption (ESA) spectra of **1** were investigated with a femtosecond transient absorption pump-probe technique.^47,48^ A commercial Ti:sapphire regenerative amplifier Legend F-1 K-HE (Coherent, Inc.) producing a pulsed laser beam with output wavelength of ≈ 800 nm, pulse energy, *E*_P_ ≈ 1 mJ, pulse duration, τ_P_ ≈ 140 fs (FWHM), and 1 kHz repetition rate was split into two parts. The first beam was converted into the second harmonic with a 1 mm BBO crystal and used as a pump beam with λ ≈ 400 nm and *E*_P_ ≤ 15 μJ. The other part of the laser beam at 800 nm was attenuated and focused into a 2 mm sapphire plate to produce a white-light continuum, which was used as a probe beam with *E*_P_ ≤ 10 nJ. The pump and probe beams were overlapped in the sample solution at a small angle, and the spectrum of the transmitted probe beam was determined with a spectrometer Acton SP2500i and CCD camera Spec-10 (Princeton Instruments, Inc.). A variable time delay between the pump and probe pulses was obtained with an optical delay line M-531.DD (PI, Ltd.) while the estimated total temporal resolution of the employed experimental setup did not exceed ≈ 300 fs. All sample solutions were placed in a 1 mm path length flow cell to reduce possible effects of photodecomposition and thermooptical distortions.

The potential for superluminescence (i.e., amplified spontaneous emission)^49^ and lasing ability of **1** were investigated in concentrated MeOH solution (C ≥ 10^–3^ M) under 1 kHz femtosecond pumping with transfer excitation geometry using the second harmonic of the regenerative amplifier Legend F-1 K-HE (λ_ex_ ≈ 400 nm). The pump beam with *E*_P_ ≤ 40 μJ was focused by a quartz cylindrical lens into a 1 cm path length spectrofluorometric quartz cuvette to a waist of 0.15 × 10 mm. The superluminescence of **1** was observed in the transverse direction relative to the pump beam and was detected with a spectrometer Acton SP2500i and CCD camera Spec-10.

### 2.3 Quantum-Chemical Analysis

The electronic properties of normal (N) and tautomer (T) forms of chromophore **F** in peptide **1** (see Figure 1) were analyzed using the Gaussian 2009 suite of programs.^50^ The linker (peptide-CH_2_)-**F** was simplified by the CH_3_ group in the model chromophore (CH_3_-**F** marked as **F_M_**). The equilibrium geometry of each tautomer form of **F_M_** in the ground state was optimized using DFT with the 6–31 G(d,p) atomic basis set and B3LYP functional. The optimized molecular geometry and corresponding properties of the excited states were obtained with TD-DFT using the same atomic basis set and functional. Linear absorption and emission transition energies, along with corresponding oscillator strengths and orbital configurations, were determined using optimized molecular geometries in the ground and first excited singlet state for absorption and emission spectra, respectively. The results of all calculations were obtained for **F_M_** in vacuo with the assumption of weak effects of the solvent environment on the energies of the electronic states of the tautomer forms of **F_M_**, as was observed for manifold 3HF and 3HC derivatives in liquid media at room temperature.^9,51−53^

## 3 Results and Discussion

### 3.1 Linear Spectroscopic Properties and Photostability of 1

**Figure 2:**
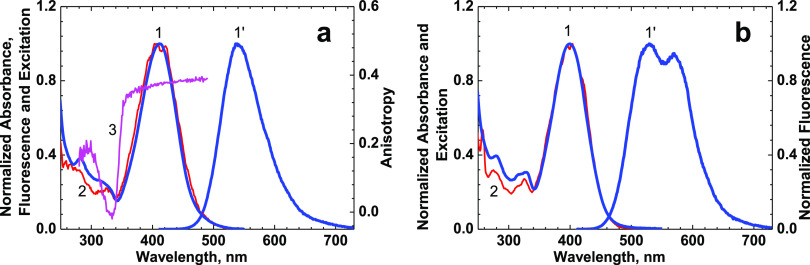
Normalized steady-state absorption (1, blue curves), fluorescence (1′, blue curves), and excitation (2, red curves) spectra of peptide **1** in MeOH (a) and ACN (b). Excitation anisotropy spectrum of **1** in glycerol ((a), curve 3, magenta).

**Figure 3:**
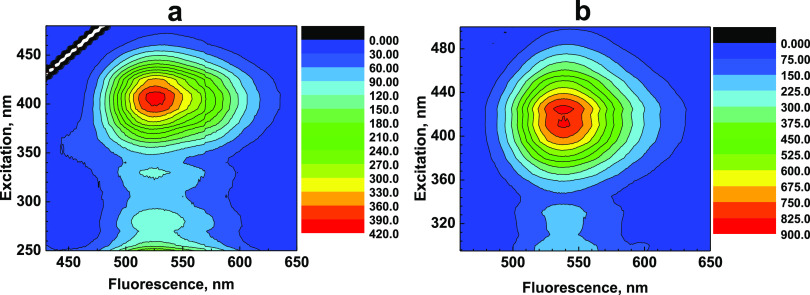
3D fluorescence emission maps of peptide **1** in ACN (a) and MeOH (b).

**Figure 4:**
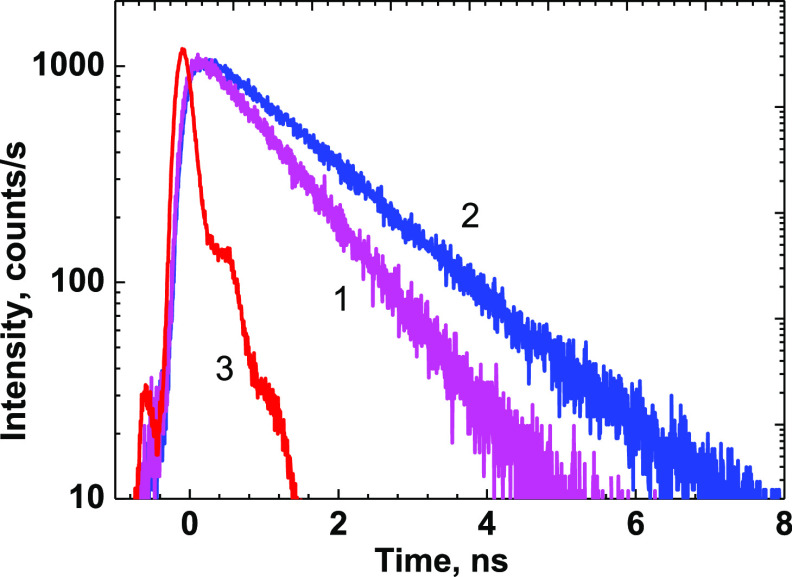
Fluorescence decay kinetics of peptide **1** in ACN (1) and MeOH (2); instrument response function (3).

**Figure 5:**
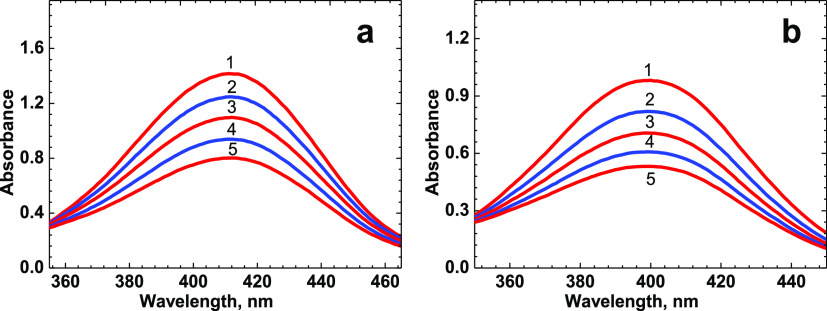
Photodecomposition spectral changes of peptide **1** in MeOH (a) and ACN (b) under irradiation at ≈ 405 nm with intensity ≈ 40 mW·cm^–2^ and corresponding irradiation times, *t*_0_ = 0 min (1) and 1–4 min (2–5).

The main linear spectral and photochemical parameters of peptide **1** are presented in Figures 2–5 and Table 1. The steady-state linear absorption spectra of **1** (Figure 2, curve 1) exhibited structureless long-wavelength absorption bands at ∼399–412 nm with relatively weak intensity (maximum extinction coefficient, ε^max^≈ (24–25)·10^3^ M^–1^ cm^–1^) and mild dependence on solvent polarity (see Table 1). Taking into account the spectral and electronic properties of similar 3HF derivatives,^9,16^ it is reasonable to assume that the observed long-wavelength bands can be assigned to the S_0_ → S_1_ transition with π → π* character^54,55^ (S_0_ and S_1_ are the ground and first excited electronic state, respectively). The absorption spectra belong to the normal (N) form of the fluorophore part (**F**) in **1** (see the molecular structure in Figure 1), which can exhibit ESIPT processes under photoexcitation.^9,14,16,51^ The steady-state fluorescence spectra of **1** revealed only one emission band with relatively large Stokes shifts (∼6000 cm^–1^) that can be assigned to the excited state tautomer form (T*) fluorescence of **F**. The emission from the excited state normal form (N*) of **F** was not observed, presumably because of relatively fast (∼ ps timescale) ESIPT processes that were frequently observed for similar 3HF derivatives.^51,55−57^ According to the 3D emission maps of **1** (Figure 3), the shape of the obtained fluorescence spectra is independent of the excitation wavelength. The fundamental anisotropy spectrum of **1**, *r*_0_(λ), was obtained in viscous glycerol solution (Figure 2, curve 3) and exhibited relatively high (≥ 0.36) and nearly constant values in the main long-wavelength absorption band. This is consistent with a sufficiently small angle between the absorption, μ_01_, and emission, μ_10_, transition dipoles of the normal and tautomer forms of **F**, respectively, and only one electronic transition, S_0_ → S_1_ in the main absorption band of **1**.^45^ The values of μ_01_ can be estimated from the experimental long-wavelength absorption contour as follows: , (where ε(*v*) is the extinction coefficient in M^–1^ cm^–1^, *v*^max^ = 1/λ_ab_^max^, and λ_ab_^max^ is the absorption maximum in cm),^58^ and corresponding data are presented in Table 1. Estimated transition dipoles μ_01_ ≈ 6.4–6.6 D are in good agreement with the results of quantum-chemical analysis presented in Section 3.3 (Table 2). Fluorescence quantum yields of **1** were practically the same in aprotic (ACN) and protic (MeOH) solvents and relatively high (Φ_fl_ ≥ 0.4) in comparison with those of its similar chromophore part (**F)** 3HF derivatives.^51,59,60^ It is worth mentioning that possible effects of hydrogen bonding processes in protic MeOH (which can dramatically change the efficiency of the ESIPT reaction^9,36,61^) did not affect the values of Φ_fl_. The steady-state excitation spectra of **1** nicely overlapped with corresponding absorption spectra (Figure 2, curves 2), and, therefore, the values of Φ_fl_ were independent of the excitation wavelength. The fluorescence emission kinetic curves of **1** exhibited a single-exponential character (Figure 4) with fluorescence lifetimes, τ_fl_, within the range of 1.1–1.5 ns and sufficiently close to each other in both solvents (see Table 1). Taking into account nearly the same values of Φ_fl_ in ACN and MeOH, similar natural radiative lifetimes^45^ of the tautomer form (T*) of **F** in the employed solvents can be assumed. All these data revealed a dominant rate of the ESIPT process in comparison with excited state radiative and nonradiative relaxations of T*. It should also be mentioned that possible reverse ESIPT processes (T* → N*)^36,59,60^ can be excluded for peptide **1** in ACN and MeOH.

**Table 1:** Main Photophysical and Photochemical Parameters of Peptide **1** in ACN and MeOH: Absorption λ_ab_^max^ and Fluorescence λ_fl_^max^ Wavelength Maxima, Stokes Shifts, Maximum Extinction Coefficients ε^max^, Calculated Transition Dipole Moments μ_01_, Fluorescence Quantum Yields Φ_fl_, Fluorescence Lifetimes τ_fl_, and Photodecomposition Quantum Yields Φ_ph_

solvent	λ_ab_^max^, nm	λ_fl_^max^, nm	Stokes shift, cm^–1^ (nm)	ε^max^ × 10^–3^, M^–1^·cm^–1^	μ_01_, D	Φ_fl_, %	τ_fl_, ns	Φ_ph_
ACN	399 ± 1	529 ± 1	6160 (130)	24 ± 2	6.4	44 ± 2	1.1	7.7·10^–4^
MeOH	411 ± 1	540 ± 1	5810 (129)	25 ± 2	6.55	40 ± 2	1.5	8.0·10^–4^

**Table 2:** Calculated Electronic Parameters of the Model Compound: Transition Wavelengths, λ, Oscillator Strengths, *f*, Transition Dipoles, μ, Transition Types, and Orbital Configurations of **F_M_** In Vacuo for the Main Transitions (HOMOs and LUMOs Represent the Highest Occupied and the Lowest Unoccupied Molecular Orbitals, Respectively)

tautomer form	transition	λ, nm	*f*	|μ|, D	transition type	main configuration
N	S_0_ → S_1_absorption	381	0.5137	6.4472	π → π*	0.98 |HOMO → LUMO>
	S_0_ → S_2_	310	0.0594	0.6056	π → π*	0.93 |HOMO-1 → LUMO
	S_0_ → S_3_	305	0.0000	0.0001	n → π*	0.98 |HOMO-4 → LUMO>
	S_1_ → S_0_fluorescence	430	0.4432	6.2735	π → π*	0.99 |HOMO → LUMO>
T	S_0_ → S_1_	499	0.5002	8.2124	π → π*	0.99 |HOMO → LUMO>
	S_0_ → S_2_	369	0.0000	0.0004	n → π*	0.97 |HOMO-2 → LUMO>
	S_0_ → S_3_	356	0.1361	1.5968	π → π*	0.94 |HOMO-1 → LUMO>
	S_1_ → S_0_fluorescence	548	0.4123	7.4321	π → π*	0.99 |HOMO → LUMO>

The investigation of the photochemical stability of **1** was performed quantitatively in air-saturated solutions using an absorption method^46^ with low-intensity laser excitation in the main long-wavelength absorption band. The observed changes in the linear absorption spectra of **1** are shown in Figure 5 for ACN and MeOH solutions under excitation at ≈ 405 nm. These data were employed for the determination of the photodecomposition quantum yields, Φ_ph_, using eq 1, and corresponding values are presented in Table 1. The analysis of the observed photodecomposition processes of **1** revealed nearly first-order kinetics^62^ and no evidence of the substantial photoproducts in the irradiated solutions at the absorption maxima. The values of Φ_ph_ were in the range of (7–8)·10^–4^ (see Table 1), which are comparable with the corresponding characteristics of laser dyes^63−65^ and acceptable for practical use.

### 3.2 Femtosecond Transient Absorption Spectroscopy and Superluminescence Properties of 1

**Figure 6:**
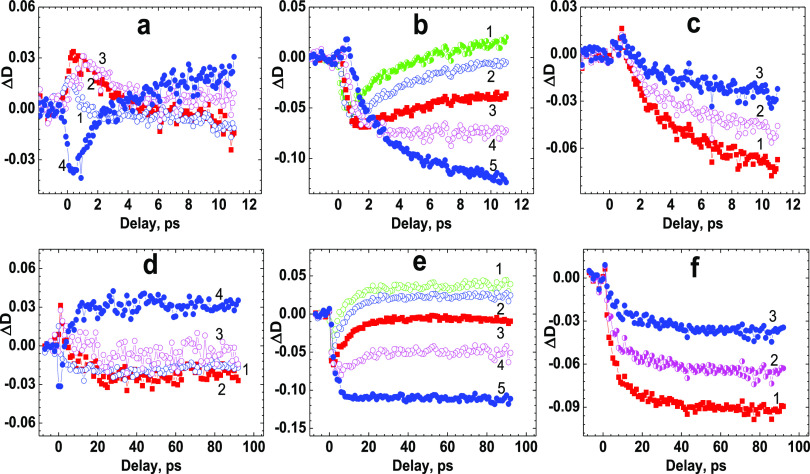
Kinetic dependences Δ*D* = *f*(τ_D_) for peptide **1** in MeOH at femtosecond (a–c) and picosecond (d–f) temporal resolution, and specific probing wavelengths: (a, d) λ_pr_= 430 nm (1, blue hollow circles), 450 nm (2, red filled squares), 460 nm (3, magenta hollow circles), and 480 nm (4, blue filled circles); (b, e) λ_pr_= 490 nm (1, green circles), 500 nm (2, blue hollow circles), 510 nm (3, red filled squares), 520 nm (4, magenta hollow circles), and 540 nm (5, blue filled circles); (c, f) λ_pr_= 580 nm (1, red filled squares), 590 nm (2, magenta circles), and 610 nm (3, blue filled circles).

**Figure 7:**
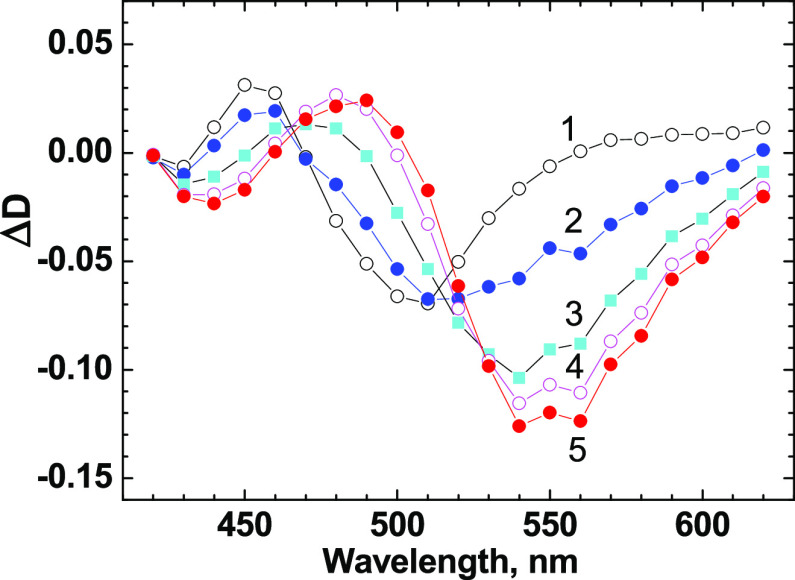
Transient absorption spectra of peptide **1** in MeOH for τ_D_ = 1 ps (1, black circles), 2 ps (2, blue circles), 5 ps (3, cyan squares), 10 ps (4, magenta circles), and 20 ps (5, red circles).

The nature of fast relaxations and time-resolved transient absorption spectra of peptide **1** were studied in air-saturated MeOH solution at room temperature by a femtosecond pump-probe technique,^47^ and corresponding data are shown in Figures 6 and 7. Temporal dependences of the induced optical density, Δ*D*, on the time delay between pump and probe pulses, τ_D_, (Figure 6a–f) were obtained over a broad spectral range (420–620 nm), and characteristic evidence of saturable absorption (SA), ESA, and optical amplification (gain) phenomena,^66,67^ including a fast ESIPT process between N and T chromophore forms in **1**, was obtained. It is worth mentioning that no direct evidence of ESIPT in peptide **1** (such as double band fluorescence emission)^21,23^ was deduced from the steady-state spectral data. All the observed transient absorption signals arise in the first ∼0.5 ps and exhibit specific behavior for different probe wavelengths, λ_pr_. Very weak negative values of Δ*D* were detected in the main absorption band of **1** at λ_pr_ ≈ 420 nm (not shown), which indicated the main role of the SA process related to the depopulation of the ground state of the N form, along with possible influence of ESA effects in the excited state of N. Relatively large short-term ESA signals were observed in the spectral range λ_pr_ ≈ 430–460 nm (Figure 6a, curves 1–3) with characteristic relaxation times of ∼1–3 ps. These signals can be interpreted as evidence of Frank–Condon and/or solvate relaxation processes^68,69^ in the excited states of the N form. The following long-term weak negative Δ*D* signals at λ_pr_ ≈ 430–460 nm gradually arose in the next ∼5–10 ps after ESA relaxation and can be attributed to dominant SA effects in the ground state of the N form. The opposite dynamics of transient absorption signals was observed at λ_pr_ ≈ 480 nm–500 nm (Figure 6a, curve 4 and 6e, curves 1, 2): short-term negative Δ*D* processes with characteristic times of ∼2–5 ps were gradually transformed into the long ESA signals in the next ∼8–10 ps (Figure 6d, curve 4 and 6e, curves 1, 2). These sufficiently intensive short negative Δ*D* signals cannot be explained by the SA phenomenon at ≈ 480–500 nm because of a weak linear absorption in this spectral range and should be attributed to gain processes from the excited states of the N form. Observed long ESA signals were nearly constant on an ∼100 ps time scale and slowly relaxed to zero in accordance with the nanosecond fluorescence kinetics of the T* form of chromophore **F** in **1**. Transient absorption curves for λ_pr_ ≈ 510–620 nm and τ_D_ ≥ 8–10 ps revealed efficient gain processes over the entire fluorescence spectral range of the T* form (Figure 6e, curves 3–5 and f, curves 1–3). These data allow estimation of the characteristic time of the ESIPT process in **F** in MeOH as ∼10 ps, which is similar to 3HF in ACN.^70^ The transient absorption spectra of **1** are shown in Figure 7 for specific values of τ_D_ and exhibit two dominant bands: the short-term (∼1–3 ps) ESA band at ∼440 nm related to the N form of chromophore **F** and long-term (> 100 ps) gain band related to the T* form. According to these data, the fluorescence contour of the T* form nicely overlapped with the observed gain profile, suggesting efficient stimulated emission properties of peptide **1**.

**Figure 8:**
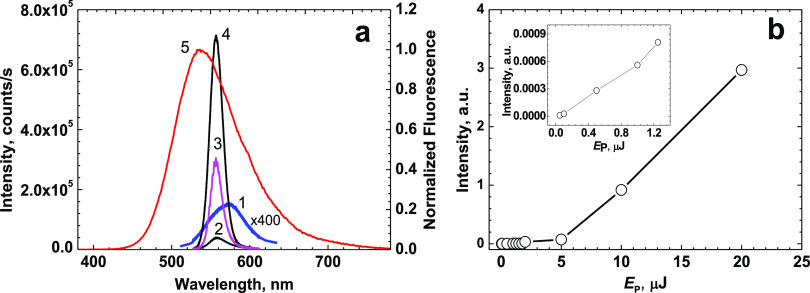
(a) Superluminescence and spontaneous emission bands of peptide **1** in MeOH (C ≈ 5·10^–3^ M) under femtosecond pumping at ≈ 400 nm with pulse energy, *E*_P_ ≈ 0.1 μJ (1, blue curve), 1.75 μJ (2, black curve), 2 μJ (3, magenta curve), 5 μJ (4, black curve), and unreabsorbed normalized fluorescence emission contour (5, red curve). (b) Corresponding dependence of the integrated emission intensity on pump energy, *I* = *f*(*E*_P_), and the initial part of this dependence (see the inset).

The superluminescence potential of **1** was estimated for a relatively concentrated MeOH solution (C ≈ 5·10^–3^ M) under femtosecond transverse pumping in the main long-wavelength absorption band at ≈ 400 nm. The spontaneous fluorescence emission spectrum of **1** was highly reabsorbed at this concentration (Figure 8a, curve 1) and consistently transformed into a relatively narrow (FWHM ∼20 nm) spectral band of superluminescence (curves 2–4) with the increase in pumping pulse energy, *E*_P_. The dependence of the collected fluorescence emission, *I*, on *E*_P_ exhibited an obvious threshold behavior (Figure 8b) with a nearly linear character for sufficiently small pulse energies (see the inset in Figure 8b) and a threshold value of ≈ 1.5 μJ. It should be mentioned that the development of superluminescent labels for bioimaging is an important step in advancements of modern fluorescence microscopy techniques.^71,72^

### 3.3 Quantum-Chemical Analysis of the Electronic Structure of 1

**Figure 9:**
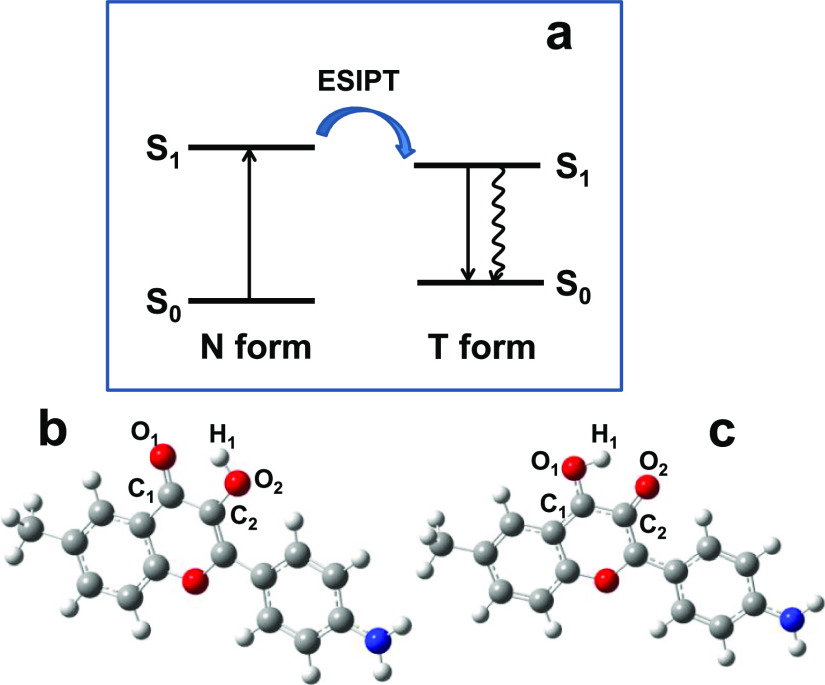
Schematic representation of the ESIPT process in **F_M_** (a). Optimized molecular geometries for the N form of **F_M_** in the S_0_ electronic state (b) and for the T form in the S_1_ state (c).

The nature of spectral properties of the tautomer forms of peptide **1** was investigated theoretically using DFT/TD-DFT calculations and the model chromophore structure, **F_M_** (see sec. 2.3 and Figure 9). Optimized molecular geometries of the N and T forms of **F_M_** in the S_0_ and S_1_ electronic states are shown in Figure 9b,c, respectively, with the indication of corresponding atoms of interest. Presented optimized molecular geometries look very similar, and the corresponding calculated bond lengths are nearly the same for both tautomers (maximum differences, Δ*l* < 0.01 Å) for all pairs of atoms except those indicated in Figure 9b,c (Δ*l* ∼0.02–0.04 Å) and mainly responsible for the ESIPT process. The main calculated electronic parameters of the N and T forms of **F_M_** are summarized in Table 2. As follows from these data, the values of absorption (N form) and fluorescence (T form) maxima, along with the transition dipoles μ_01_, are nicely correlated with the corresponding experimental parameters of peptide **1** (see Table 1). Calculated components of the transition dipoles μ_01_ for S_0_ → S_1_ (λ= 381 nm; μ_01_^*x*^= 4.109 D; μ_01_^*y*^ = −4.968 D; and μ_01_^*z*^ = 0) and μ_02_ for S_0_ → S_2_ (λ= 310 nm; μ_02_^*x*^= 0.119 D; μ_02_^*y*^= 0.594 D; and μ_02_^*z*^= 0) electronic transitions reveal a sufficiently large angle (≈ 51°) between the vectors μ_01_ and μ_02_. This value is close to the magic angle (54.7°)^45^ and gives nearly zero anisotropy in the case of collinear orientation of μ_01_ (N form) and μ_10_ (T* form). These data are in a good agreement with the experimentally obtained excitation anisotropy spectrum of peptide **1** (see Figure 2a, curve 3), where the minimum near zero value is observed at ≈ 330 nm.

## 4 Conclusions

Linear photophysical and photochemical properties, fast relaxation processes, and stimulated emission of new fluorescent peptide **1** were comprehensively investigated in liquid media at room temperature. The steady-state absorption and fluorescence spectra of **1** revealed a relatively large Stokes shift (∼6000 cm^–1^), only one emission band with lifetime ∼1.1–1.5 ns, and a quantum yield of ≈ 0.4 that can be associated with the fast ESIPT process. Femtosecond transient absorption spectroscopy of **1** directly confirmed that the ESIPT process was operative with a characteristic time of ∼10 ps, without noticeable reverse transformation, and optical amplification in the fluorescence spectral range. An efficient ESIPT-based superluminescence phenomenon was observed for **1** in MeOH under one-photon femtosecond pumping, a photophysical process that is important for the development of new fluorescent labels with increased spectral brightness. DFT/TD-DFT quantum-chemical calculations were performed to analyze the electronic structure of the fluorescent chromophore in peptide **1** and were in good agreement with experimentally observed properties. The spectroscopic data of a new fluorescently labeled peptide, including steady-state and time-resolved emission properties, fast ESIPT, and efficient superluminescence processes, reveal the potential for its application in a number of important multidisciplinary areas, such as laser scanning fluorescence microscopy, environmental monitoring, and biomedical diagnostics.
